# Introductory Address on the Opening of the Medical Department of Lind University

**Published:** 1860-01

**Authors:** N. S. Davis

**Affiliations:** On the opening of the Medical Department of Lind University


// THE CHICAGO
### MEDICAL EXAMINER

Vol. I. JANUARY, 1860. No. 1.

### Introductory Address by Prof. N. S. Davis, on the opening of the Medical Department of Lind University.

*Members of the Medical Class and Fellow Citizens:*

The occasion on which we are assembled is one of no ordinary interest. The intimate relations which the medical profession bear to some of the most important and most sacred interests of human society, make everything relating to its education a matter of deep public concern. Hence we are gratified to see before us, not only the officers and trustees of the University, with the members of the medical class, but also many of our most enlightened citizens. We are assembled at the present time, not merely to open an ordinary college term, but to dedicate a new institution, and formally consecrate its halls to the noble purpose of diffusing a knowledge to the science and art of medicine—a purpose second in importance to no other of a temporal nature. It is not, however, merely the opening of a new institution, the addition of one more to the number of medical colleges already existing in our country, that has called us together this evening; but the opening of one on a different and, we humbly trust, better plan than any which have preceded it on this side of the Atlantic. Having thus deviated from the beaten path, the strict line of precedents in the establishment of this department of the Lind University, it may be reasonably expected that we will embrace the present opportunity to develop, so far as the hour allotted to us will permit, the reasons by which we have been influenced, the nature and extent of the changes we have adopted, and the objects we propose to accomplish by them.

The considerations which have induced the faculty to undertake the task of establishing this institution, may all be included in the two following propositions :

First, the very liberal offer of the Board of Trustees of the University, to furnish all the needed accommodations for a medical department, with no other restrictions than that the plan of instruction adopted should be such as would most effectually promote the educational interests of the profession without reference to established customs and usages.

Second, a sincere desire on the part of the faculty to put into practical operation a system of medical college instruction more in accordance with sound educational principles, and better adapted to the present state of the science and art of medicine, than that which has been so long adhered to by the medical schools of this country.

As this last proposition rests upon the assumption that the present system of medical college instruction in this country is defective, it may be proper to spend a few moments in investigating the truthfulness of that assumption, more especially as without this we might be charged with personal arrogance or an attempt to be wiser than our generation. It is well known that the system of medical education in vogue in this country has been the subject of discussion and severe criticism for many years. These discussions in the medical periodicals, and the State and local medical societies, led to the assembling of a National convention of delegates from the various medical societies and colleges in the United States, at New York, in May, 1846, and to an adjourned convention in Philadelphia the following year, when a permanent National Association was organized. At the primary convention in New York, three committees were appointed having reference to the subject of medical education.

The first was to report on the subject of preliminary education ; the second, on the requirements necessary for graduation ; and the third, on the proposition to separate from the colleges the right to issue diplomas which confer the right to practice.

Carefully considered reports were received from each of these committees, at the meeting in Philadelphia the succeeding year. The first was presented by Dr. Couper, of Delaware, chairman of the committee, and strongly urged upon both the profession and the colleges the adoption of a higher standard of preliminary education before entering upon the study of medicine. The report from the second committee was presented by Dr. Haxall, of Virginia, and not only admitted that there were many and important defects in the prevalent system of college instruction, but specifically recommended an increase in the length of the annual college terms, an increase in the number of professors, an extension in the curriculum of study, and the exaction of a higher standard of qualification on the final examination for the degree. Both these reports were accepted by the convention, and after a free discussion, their recommendations were adopted with great unanimity. Two reports were received from the third committee, both admitting the system of Medical College instruction to be defective, but without definite action they were referred to the standing committee on medical education for the ensuing year. At the annual meeting of the American Medical Association in Baltimore, the following year, (1848,) the report from the standing committee on medical education was made by Dr. Alexander H. Stevens of New York, himself one of the oldest and ablest teachers in the Union. In his report he uses the following language, viz: "The truth of the proposition that there are striking deficiencies in our profession is, at this time, so generally conceded as to obviate the necessity of further demonstration." And again he says: "In whatever aspect the enterprise be viewed, the mind is finally arrested by the apparently radical source of all the evils and deficiencies in the profession, viz: the imperfect education of a large part of its members." At the close of his report Dr. Stevens urged a strict observance of the recommendations adopted the previous year, and in addition that hospital clinical instruction be made a necessary part of the Medical College instruction, and that the "faculties of medical schools be advised and requested, carefully to examine students after attendance on their first course of lectures, to issue certificates of proficiency to those who merit them, and to regard the possession of such certificate, and attendance on another course of lectures indispensable preliminaries to a final examination for the doctorate."

At the next annual meeting of the American Medical Association, which was held in Boston, in May, 1849, and which was very fully attended by members and delegates from every part of our Union, the report on medical education was made by Dr. F. Campbell Stewart, of New York, chairman of the standing committee on that subject. This report contains an elaborate and highly interesting review of the system of medical college instruction adopted in the several European countries; and after comparing them with that existing in our own country, the author says: "The subjects taught in Europe are more numerous, and a much greater proportion of time is devoted to their study than is allowed in the United States. They are so disposed, also, that they follow each other in a regular consecutive order. The student is thus enabled to prepare himself on a given number of subjects, by close application to their details, in a reasonable period of time; after which he is examined; and, if successful, his mind being relieved of a portion of care and anxiety, he is better prepared to commence and prosecute the study of a new series, upon which he is likewise in turn examined, and which he dismisses for the time being from his thoughts. How infinitely superior is this course to that which compells him to burden his memory, and toil during the whole period of his attendance on lectures, to keep pace with his preceptors in their endeavors to impart to him instruction on a multitude of subjects, which are crowded together, and a knowledge of all of which must be obtained in the very short space of time allowed by most of our colleges as the period in which their courses are comprised."

Again, Dr. Stewart says: "The number of professors engaged in teaching medicine and surgery in connection with European schools is more than double, and, in some instances, four and five times as great as with us. This permits a division of labor, and enables those engaged in imparting knowledge to devote their time and energies to the full and comprehensive illustration of the subjects, the elucidation of which has been committed to them."

Besides reiterating the principal recommendations made in the reports of previous years, we find in this the following important propositions, viz: "We think that much might be gained by a division of the subjects taught into two classes; one series of which might be studied during the first course of lectures, and another during the second year's attendance. The anatomy and dissecting, together with chemistry, materia medica, pharmacy and physiology, might be studied during the first session, at the close of which, examinations should be held and certificates of acquirement given. During the second session, the subjects of surgery, practice of medicine, midwifery, and hospital attendance, with a continuation of the study of anatomy, might be insisted on. This we think would be a decided improvement upon the present plan, which requires attendance on all the branches during both sessions, and does not permit the student time to prepare himself thoroughly on any one of them. We urge your close attention to this proposition, which we hold to be important, and which we think would be found to work well."

At the same annual meeting of the Association, a special committee was appointed to embody in a report more fully the views of the profession on the subject of increasing the length of the annual lecture term. This committee consisted of the venerable Dr. Samuel Jackson, as chairman, and Drs. J. L. Atlee and Alfred Stille, all of Philadelphia. In the very able report of that committee we find it stated that, "to the imperfect and restricted courses of the schools, and the low standard of medical graduation, were attributed the superficiality and degradation of medicine." Again: the plan of four month courses of lectures belongs to the origin of medical schools in this country, and arose out of the necessities of the case. The establishment of medical lectures at all was a bold innovation; and, lest it might act as a discouragement to students, the term was made as short as possible, and limited to four months. And yet, at that period, medicine had but a moderate expansion, and scarcely made pretension to a scientific character."

The same report continues: "Since the first establishment of the medical schools, the field of medical science has changed its entire aspect. The new departments that have been developed, exceed in extent, and equal in importance, the rudimentary branches forming the original scheme of medical education. They embrace what may be correctly designated the higher and scientific branches of education. To include them with the original courses, in lectures of four months' duration, is wholly impossible." After speaking of the crowded state of the profession, and its imperfect education, the same committee add : "The profession look to the schools to reform this evil; and they anticipate longer courses, new branches added, higher requisites for graduation, and an adequate preliminary education, as the means by which it is to be accomplished."

Elaborate and able reports from the standing committee on medical education were made to the Association, at its annual meetings in 1853, by Dr. Worthington Hooker, of Connecticut; in 1854, by Dr. G. L. Cabell, of Virginia; in 1857, by Dr. W. H. Anderson, of Alabama. In each of these reports the defective condition of our medical college courses of instruction was as fully acknowledged as in any that we have already noticed.

The last report which has been made to the Association on this subject, emanated from a special committee, of which Dr. James R. Wood, of New York, was chairman. The committee was appointed at the annual meeting of the Association in Nashville, May, 1857, and was instructed to report to the next annual meeting a definite plan of medical college organization and instruction. At the succeeding meeting in the city of Washington, May, 1858, Dr. J. R. Wood presented his report, which recognized the inadequacy of the prevailing system of medical college instruction, in the following explicit language, viz: "The great advancement of medical science during the last few years has materially changed the character of the curriculum of medical studies. The more common branches, as anatomy, chemistry, practical medicine, surgery, obstetrics, and materia medica, have been indefinitely enlarged, and now require for their complete elucidation far more time, and more patient and painstaking demonstration. But, in addition to the vast improvements, and their consequent expansion, other fields in the domain of medical science have been opened for investigation, and earnest, thoughtful laborers have cultivated them not in vain. To afford the student facilities, therefore, for obtaining a complete and thorough medical education, our schools must increase the number of their professorships, in proportion as each new department of medical science attains the rank of a definite science.

Every teacher of medicine must be impressed with the importance of giving to both teacher and pupil more time, not only by lengthening the terms of our colleges, but also by having fewer lectures daily. The system, as at present pursued, is literally one of "cramming," and must sooner or later be essentially modified."

We have made these copious, and perhaps tedious extracts, not from the statements of those who are styled special advocates of reform in medicine, nor from public addresses, where the excitement of the occasion or the superfluities of rhetoric might lead to exaggerated expression; but from reports deliberately prepared by committees, after ample time for investigation, and the sentiments of which have been repeatedly sanctioned by the highest authority known to our profession, viz: the American Medical Association. They not only fully justify the assumption on which we have acted, but they show, clear as the noon-day sun, the necessity for a system of medical college instruction more comprehensive and systematic than that which has hitherto prevailed in our country. Hence, instead of seeking to excuse ourselves for having embraced the opportunity presented by the enlightened board of trustees of this University, to establish a medical school on a broader basis, with a more extended and systematic plan of instruction, we are free to acknowledge that any other course on our part would have proved us recreant alike to the interests of the profession and the great cause of humanity.

The extracts which we have made from the records of the National Medical Association, not only show the defectiveness of the prevalent system of medical college instruction, but they indicate in general terms the appropriate remedies or improvements that are desired. They are, first, an increase in the number of professorships corresponding with the increased number and extent of the branches included in the great field of medical science and art, at the present time. Second, an increase in the length of the lecture term sufficient to allow fewer lectures a day and the students more time for reflection and hospital attendance. Third, such a division of the branches as will enable the student to attend, during the first course of lectures, to those only which are more elementary in their nature ; and in his second course, those denominated practical; thereby enabling him to concentrate the mind upon a smaller number of subjects at one time, and investigate them in such order of succession as will facilitate both the acquisition of knowledge and the attainment of a high degree of mental discipline. Fourth, the establishment of systematic hospital clinical instruction in connection with the courses on practical medicine and surgery. Fifth, the more frequent and thorough examination of students during their attendance on lectures, as well as at the close of the period of their pupilage. In devising a plan of instruction for this department of the University, we have not been unmindful of these deliberately expressed sentiments of the profession. On the contrary, we have studiously endeavored to execute such a plan of organization as would insure their complete practical accomplishment. To make the peculiarities of this plan obvious, it is necessary to state cursorily the principal features of the ordinary medical college courses, with an explanation of the actual relations which the colleges bear to the entire education of the medical student. From the earliest organization of medical colleges in our country, their active period of instruction has been limited to a part of each year, and consists almost entirely in oral instruction by lectures, aided by such demonstrations as the several branches will permit. Until within a very few years, nearly all the colleges commenced their annual courses of instruction about the first of November, and continued sixteen weeks. In a few schools, the entire annual course of instruction embraced only thirteen weeks. Within the last three years, owing to the repeated recommendations of the profession, through the American Medical Association, several of the colleges have added two weeks to the length of the term, by commencing the second on third week in October, instead of the first of November. The usual number of professors in each school is seven; and the number of lectures each day six, except in such as devote a part of two days in each week to clinical purposes. In only a part of the schools is hospital clinical instruction and dissections made a necessary part of the curriculum of study. It will thus be seen that the whole field of medical college instruction is annually crowded into the short space of from fifteen to eighteen weeks—that the student is required to listen to an average of six lectures per day, on as many different subjects—that these subjects are presented in no natural order of succession, but heterogeniously embracing the same day, lectures on anatomy, chemistry, materia medica, practical medicine, surgery, and obstetrics—that no division of the several courses, so as to adapt them to the period of advancement of the student is allowed; but the student who has studied medicine less than six months, and is yet scarcely familiar with the frame work of the human system, or the natural functions of its most important organs, is required to listen day by day to the same details on practical medicine, surgery, and obstetrics, as the student who has been diligently pursuing his medical studies for three years.

Such are the principal features of the prevalent system of medical college instruction in our country. To show its inadequate extent, and its violation of the plainest and most important educational principles, we need only refer to a few facts.

For instance, no enlightened physician would regard a course of medical study as sufficient, which did not include anatomy, physiology, histology, chemistry, both organic and inorganic, materia medica, general pathology, surgical and pathological anatomy, medical jurisprudence and toxicology, practical medicine, practical surgery, and midwifery, with diseases peculiar to women and children. Yet a single standard work on each of these branches would embrace not less than seventeen large octavo volumes, averaging over 600 pages each, or an aggregate of over 10,000 printed pages.

It is quite obvious that any attempt to distribute this whole field among six or seven professors, for the purpose of bringing it under review in sixteen weeks, is a practical impossibility.

On this subject the profession of Ohio, assembled in convention at Columbus, in January, 1838, passed two resolutions as follows:

"*Resolved*, That in the opinion of this convention, the sessions of the different medical schools throughout this Union are too short, and that they ought to be extended one month, and the students required to stay to the end of the term.

"*Resolved*, That the number of professorships is too few, and that ampler provision be made for teaching physiology, pathological anatomy, pharmacy, medical jurisprudence" &c.

Dr. Daniel Drake, (than whom no higher authority can be quoted in the interior of this continent,) in commenting on these resolutions in the Western Journal of Medical and Physical Sciences, for March, 1838, says: "Their first resolution, however, contains suggestions in which every reflecting member of the profession must concur. That the lecture terms, in all the schools in the Union, are too short, is undeniable." After censuring severely the practice prevailing among students of leaving the college before the term is finished, and suggesting that all students should be required to take "a ticket of valediction," as well as of matriculation, Dr. Drake continues: "We cannot but cherish a hope, that this regulation, together with an extension of the term, will, at no distant day, be adopted by all our schools. The effect on the American profession would be instantaneous, and, in all respects, salutary."

Again he says: "The second resolution furnishes a strong argument in support of the first, and ought, indeed, to have preceded it in the series. It looks directly to the limited range of studies prescribed and pursued in nearly every school in the Union. Indeed, we may affirm, that there is not one in which the cycle is as comprehensive as the nature of the medical profession demands." If these resolutions of the State medical convention of Ohio, and the observations of Dr. Drake thereon were founded in truth, when made twenty years since, how much more applicable are they now, when almost every branch of medical science has been greatly enlarged, without any material enlargement of the college courses of study.

We have intimated that the prevalent system of medical college instruction is not only too short and too limited, but that its arrangement violates the plainest and most important principles which should govern man in the acquisition of knowledge. It does this in three ways: First, by crowding upon the mind daily so great a number of diverse and intricate topics, that it is impossible to bestow any reasonable amount of reflection on each: Second, by presenting the several branches in a perfectly heterogenous manner, without any regard to their natural relations to each other; and, third, by giving the same kind and amount of instruction to all the students, without any reference to their previous attainments or degree of advancement in their professional studies.

If a good student in a literary college finds his time fully occupied in endeavoring to acquire, simultaneously, a knowledge of one branch of a natural science, one of mathematics, and two languages, what shall we think of the system which requires the young man to keep pace with six lectures per day on as many different branches of medicine, with dissections in practical anatomy, and more or less clinical instruction added? It is a fundamental principle, constantly acted on in all schools, except those devoted to medical studies, that whenever a number of studies, or branches of study, are to be pursued, such as are most elementary and best calculated to prepare the mind for the others, are taken up first, and the more abstruse and complex ones afterwards. Thus grammer precedes rhetoric, and arithmetic the higher branches of mathematics, etc. Equally so in medicine, the study of anatomy and physiology, which embrace a knowledge of the structure and functions of the human system in health, should precede the study of disease, which is a deviation from health. In like manner, chemistry and materia medica, which reveal to us the composition and properties of medicines, should go before any attempt to acquire a knowledge of the application of these agents in the treatment of disease. Yet obvious, as is this principle to the sense of every man, it is, as we have already seen, entirely ignored in the system of instruction adopted by our medical schools. On these points, Dr. Drake, in the article to which we have already alluded, makes some observations so pertinent, that we will not withhold them. The third resolution adopted by the medical convention of Ohio, in 1838, was as follows:

"*Resolved*, That if practicable, our medical schools should be so organized, as that students in their first course should have their attention chiefly directed upon special anatomy, physiology, chemistry, pharmacy, and other elementary branches; and their second, upon pathological anatomy, therapeutics, the practice of physic, surgery and obstetrics."

Commenting on this resolution, Dr. Drake says: "It is not only absurd, but actually injurious, for the student who has recently commenced the study of medicine, and is not yet acquainted with the structure and functions of the body, with chemistry, or the rudiments of botany or zoology, to engage the high and difficult inquiries of pathology and practical medicine; and, in the present organization of our schools, this is constantly done. The *beau ideal* of collegiate medical instruction would be for students, in their first course, to devote themselves to anatomy, special, general and pathological, with dissections; to physiology, corporeal and mental; to chemistry, pharmacy, and the classifications of medicines; and to so much of the history of the mineral, vegetable and animal kingdoms as is necessary to the due understanding of the two last; and in the second session to give their chief attention to therapeutics, symtomatology, ætiology, practice, surgery and obstetrics."

Again he says: "It is greatly to be regretted that private preceptors do not confine the reading of their pupils, in the early period of their studies, to the introductory branches, and send them, as soon as they have taken a bird's eye view, and become somewhat familiar with technical terms, to a medical school, with instructions to limit themselves to the lectures which are proper for the first session. After attending it, they should engage in a course of more practical reading, and then return to the University for graduation. It is to be feared, however, that for a long time to come, our brethren who do not reside in the immediate neighborhood of medical schools will think, or at least act differently from what is here advised; and equally to be apprehended, that those who prescribe the policy of our institutions, will neglect the establishment of junior and senior classes. Meanwhile, we will hope, however, that the students themselves will become more and more impressed with the importance of devoting the first session chiefly to the elementary branches, and the second to the practical." Having presented clearly, and illustrated perhaps tediously, the evils and defects inherent in the organization of medical schools in this country, we will proceed to state briefly such peculiarities in the organization of the medical department of this University, as are designed to obviate these evils and defects.

They are: first, the extension of the annual college term to five months; second, the increase in the number of professorships corresponding with the number and extent of the branches actually included within the domain of modern medicine; third, the division of the term into junior and senior departments in such a way that all students attending their first course can concentrate their attention upon the more elementary branches, and advance in their second course to the more practical; fourth, the giving of fewer lectures each day, with daily examinations, and general examinations at the close of each department, thereby ensuring a much higher degree of mental discipline, and a more perfect knowledge of each branch brought under review; fifth, the elevation of clinical medicine and surgery to the rank of professorships, and the making of daily clinical instruction in the wards of a hospital a necessary part of the course in the senior department.

By these arrangements we secure to the student of medicine the means for pursuing the different branches of medical study, in a strictly methodical and natural order of succession. In the junior course, which embraces anatomy, physiology, and histology, inorganic chemistry, materia medica, and general pathology, with dissections, he concentrates his attention upon, and becomes familiar with the elementary parts of our noble science. He becomes familiar with the composition, mode of development, structural relations, and functions of the various parts of the human system in a healthy condition, together with its relations to inorganic matter, and the composition and properties of remedial agents.

He thus lays deep, broad, and well defined, the foundation of his professional education before he attempts to mingle with it the superstructure. Having done this, he advances with ease and readiness to his senior course, embracing a review of his anatomy in its relations to operative surgery: of chemistry in its application to organic matter and toxicology; and of practical medicine, surgery, obstetrics, and medical jurisprudence.

Another advantage of paramount importance to the medical man necessarily results from this plan. By taking up the branches in their natural order of succession—by concentrating the attention on a smaller number of lectures each day, thereby allowing time to reflect upon and mentally digest what is heard—and by giving each teacher fifteen minutes additional time daily, to examine the class on the lecture of the preceding day, the student almost necessarily acquires a clearness of thought and expression, a quickness of perception, and a general mental discipline of the highest value in the practice of the healing art. Without method there can be no true mental discipline. And without a good degree of mental discipline the accumulation of facts only convert the mind into a storehouse of heterogenious materials, without the ability to perceive the relations they bear to each other, or the applications of which they are capable in the investigation and treatment of disease.

Finally, this plan makes the entire course of college instruction embrace a much more complete and comprehensive review of the field of medical science and practice as it now exists.

It is well known that in the ordinary arrangements of the schools with six or seven professors, the important departments of surgical anatomy, histology, organic chemistry, general pathology, and medical jurisprudence, are each appended to other branches or entirely omitted from the curriculum. And it is equally well known that in a large majority of the schools the branches to which they are appended receive the entire attention of the teacher, or if they are reached at all, it is only in time to furnish the matter for four or five lectures at the end of the term, after half of the class have returned to their homes. Some of the students whom I now address, have attended full courses of lectures in other schools, with the or dinary number of professors, and I would inquire of them how many lectures they even listened to on the important topics of organic chemistry, toxicology, histology, and medical jurispru dence? The omission of these is not the fault of the teachers, but of the system under which they act. If to one professor is assigned both physiology and general pathology, and only four lectures per week for sixteen weeks, it is not possible for him to give more than an adequate view of the first, if he includes with it, as he should, histology, and more or less examinations with the microscope. One of the most popular text books on human physiology, now in use, contains over 1,000 large sized and closely printed octavo pages.

Again, if medical jurisprudence is appended to the chair of materia medica, as is often the case, the lecturer must possess a very unusual power of condensation, or he will find himself at the end of the sixteen weeks before he has completed his course on the latter alone. Hence we make no new or exaggerated statement when we say that the ordinary plan of medical college instruction, embracing seven professorships, and sixteen or seventeen weeks of lecturing, absolutely necessitates one of two important evils, viz: either the entire omission of several important branches of medical science, or a very hasty and inadequate presentation of the whole. But by our plan of nearly doubling the number of professorships, and dividing the annual term of five months into two distinct departments, we are enabled to embrace all the branches of medical science proper, to present each with a degree of fullness proportionate to its importance, and thereby lead the student who attends his courses with us over a much more comprehensive field of study. This is more clearly demonstrated by the following figures, viz: The student attending an ordinary college course of sixteen weeks, with six lectures per day, except Saturday afternoons, would listen to an aggregate of 520 lectures. If he attends a second course in the same school, he simply listens to a repetition of the first. Hence the 520 lectures actually embraces the entire field of study brought under review in the ordinary prevalent system of medical college instruction. The student, however, who attends his first course in the junior department of this University, receives, besides dissections and demonstrations with the microscope, four lectures per day for full twenty weeks, (omitting the afternoon of Saturday,) making an aggregate of 446 lectures on five fundamental and important branches of medical science. In his second course in the senior department of this institution he would receive four lectures per day in the college and one in the hospital, for twenty weeks, making an aggregate of 600 lectures, none of which will be a repetition of those listened to in his junior course. Hence, in attending two courses in the medical department of this University, he is conducted over a field actually embracing an aggregate of 1,040 lectures, being thus just double the extent of that passed over in the ordinary plan.

A fair comparison of the two systems of medical college instruction then stands thus: By the ordinary plan, the student attending his first course, is crowded with six lectures per day, on as many different topics, for sixteen weeks. In the second course he endures a repetition of the same process over precisely the same field.

By the plan adopted in the medical department of this University, the student in the junior department receive four lectures per day for twenty weeks, thus giving him time to reflect upon and digest what he hears, and alse pursue practical anatomy by dissection and microscopic examinations without haste and confusion.

In his second course, in the senior department; he advances to another series of branches, and receives five lectures per day for twenty weeks including clinical medicine and surgery. Can any intelligent physician or student hesitate in deciding which system or plan of instruction is most comprehensive, most systematic, and most in accordance with the plainest principles of education?

We are aware that two medical journalists have recently published the statement that "if the student is to depend on the schools for his education, a single course of lectures on any branch of science is not sufficient," but the same should be repeated once or more. We freely admit the abstract truth of the proposition; and yet it constitutes no objection to the plan of instruction adopted in this institution—simply because in no part of America does the medical student depend wholly on the schools for his education. On the contrary, the period of medical study universally claimed for the student is three years, or thirty-six calendar months; while the aggregate amount of attendance required in the schools is only eight months, or less than one-fourth of the whole.

What, then, is the true relation borne by the schools to the education of the profession in this country? Most obviously it is this: The student is to lay the foundation of his education by a careful reading of approved authors under the direction of a private preceptor, while he resorts to the schools for the purpose of hearing the several branches reviewed, accompanied by such illustrations and demonstrations as can be given by the living teacher only. Admitting this to be the actual relation of the schools to the education of the student, is there an inteligent physician who will hazard his reputation for sagacity by claiming that the schools should be so organized as to compel the student who, during the first part of his period of study, has hardly had time to read the ordinary text books on chemistry, anatomy, materia medica and physiology, to listen to a review, not only of these branches, but in addition, also, to practical medicine, surgery, obstetrics, etc., and all in the short space of four months, for the sake of having the same confused repetition at the end of the last half of his period of pupilage? With as much propriety might we require a class of boys in a grammar school, who had studied only grammar, geography and arithmetic during the year, to review at its close, rhetoric, astronomy and algebra.

But we have already wearied your patience on this subject. We have presented before you abundant testimony, from sources that can neither be gainsayed nor refuted, to prove the prevalent system of Medical College instruction extremely defective, and inadequate to the wants of the profession. We have shown from the same authoritative sources that the principles embraced in the organization of the Medical Department of this University are neither the developments of to-day, nor the invention of some over zealous partisan reformer. But on the contrary, that every principle embraced in the organization has been fully evolved and urged upon the attention of the medical public by the master minds of the profession for more than a quarter of a century. They have been, singly and collectively endorsed, not merely by State and local medical societies, but by the highest tribunal known to the profession in this country. Feeling, therefore, the fullest confidence in the correctness, both of the principles and the details involved in the organization of this institution, my colleagues and myself enter upon the task of giving it a practical establishment with no trembling hand or faltering step. On the contrary, eschewing all partisan strife and mere groveling rivalry with existing institutions, and fully conscious of the purity of our motives, and the high and noble purposes to which we have dedicated our labor, we boldly unfurl our banner to the breeze, not doubting but the time will come when the wise and good will rally under its folds from every grove and prairie in these great and fertile States of the North-west.

Another inquiry of no less interest to the profession, and especially to you, gentlemen, who have assembled here to receive instruction, is: What means do we possess for carrying into successful operation the plan of organization which we have just passed in review?

We are happy to be able to respond that they are ample in every department. Rooms have been provided in this magnificent block of buildings, furnished with all the comforts and conveniences usually found in the best colleges. They consist of two convenient, comfortable and well lighted lecture rooms, a laboratory, museum, room for practical anatomy, a library, and faculty room. The laboratory is furnished with an entirely new apparatus, selected with especial reference to illustrating a full course of instruction in each department of chemistry.

The museum is already furnished with a better cabinet of preparations and drawings, anatomical, pathological, microscopic and obstetrical, than is to be found in any other medical institution in the Northwest. And on the arrival of our colleague, the professor of anatomy, a few days hence, it will receive a large and most valuable addition, directly from the great emporium of medical science on the continent of Europe. A library has also been provided, consisting of between four and five hundred volumes, which will be accessible to the class under proper regulations.

In the all important departments of practical medicine and surgery, the means of illustration are even more complete. The Mercy hospital, located near at hand, being scarcely ten minutes walk from the College, has constituted a genuine clinical school for the last eight years. It contains about sixty beds for the sick, and always has in its wards a sufficient number of patients of both sexes, to illustrate fully, all the more important and severe forms of disease, both medical and surgical.

In addition, the Orphan asylum, immediately adjoining the hospital, furnishes the clinical class frequent opportunities for observing the diseases of children, an advantage of great value to the student, and rarely enjoyed in connection with public institutions. The hospital is open for clinical instruction to all regular students who have arrived at the proper period of advancement in their studies, from eight to nine o'clock every morning, except Sunday. We say open to all students of legitimate medicine, without reference to what college they may be attending; for though its wards are fully under the control of the professors of practical medicine and surgery in this institution, we should deem it alike illiberal and unprofessional to restrict its advantages to our own students.

On the contrary, the clinical advantages of every public hospital belong to the educational interests of the profession at large. And the physician or surgeon who would restrict them entirely to his own private interests, or to the interests of the particular school with which he might be connected, is an enemy to the profession and the cause of humanity. The clinical instruction in the hospital is of the most practical and particular character. The student is enabled to come in direct contact with the patients, and not only to note all the ordinary symptoms, but to daily train his own ear and touch by the direct practice of auscultation, percussion, palpation, etc. Two surgical cliniques are given each week, namely, on Monday and Saturday mornings, while the four intermediate mornings are devoted to clinical instruction in the medical wards.

In addition to the hospital, we have the Chicago City Dispensary, now occupying one of the rooms in this building, to which a considerable number of patients resort daily.

From these we shall select the cases of interest for a regular surgical clinique in this room, on Wednesday of each week, by the professor of surgery; and a medical clinique every Saturday by the professor of practical medicine. These cliniques in the college will be given from two to three o'clock, on the afternoons of Wednesdays and Saturdays, throughout the term, and will be free for both the attendance of the junior and the senior classes, and also any member of the profession who may choose to honor us with their visits.

Such, gentlemen, are the means at our command for carrying on the several courses of instruction provided for in the organization of this institution. On the ability of the several members of the faculty to use these means skillfully, and discharge the duties devolved upon them satisfactorily, it does not become me to speak. Of the ten active members of the faculty, eight are, and have been for several years, residents of this city; and are all well known to you, as well as to the whole profession of the northwest. Concerning the two non-residents, it may be proper to say a few words. The professor of pathology and public hygiene, a resident of Galesburg, in this State, is a gentleman of high scientific attainments, and a physician of experience, and well qualified to do honor to the chair he occupies.

The professor of Anatomy, Dr. Titus Deville, has been a resident of Paris, in France, during the last five or six years, where he has attained the reputation of being one of the best teachers of Anatomy in that great medical metropolis. He comes to our city for permanent residence, and brings with him a full supply of everything which can contribute to make the important department of Anatomy fully understood. We predict for him a success in that department which has rarely been equalled.

With an organization so methodical and comprehensive as we have detailed—with the means of carrying it into effect so ample—without a single dollar of indebtedness to embarrass its operations—and with a faculty wholly independent of any income to be derived from the institution for their own support, we may safely assume that the success and permanence of the Medical Department of Lind University is secure. We are happy to announce that there are already students enough before me, whose names have been given in for attendance during the whole term, to constitute a respectable class in each department.

Young gentlemen, we not only welcome you cordially to the halls of this institution, which we here this evening dedicate to the noble purpose of defusing a knowledge of the most interesting sciences, and the most beneficent profession that exists among men, but we congratulate you on the peculiar advantages of your position.

For if the classes in each department should remain small, compared with those attending some of the older schools, instead of operating as a discouragement, it would be, to you, a very great advantage by enabling each of you to receive that minute and thorough personal instruction in every branch of medicine which would be impossible in a class numbering from one to five hundred. Again, in after years, when the institution has attained the position to which it is surely destined, and is every where acknowledged as the pioneer in the great work of extending and elevating the most important educational interests of our profession, you will feel a just pride in the rememberance that you constituted its first class, and by your presence aided us to usher it into existence. In choosing the profession of medicine as your calling, you have individually assumed a high responsibility. Your future lives must be a continuous conflict with disease, and the grim monster, death. The fond father will often extend to you his feverish hand, imploring to be restored to health and the care of his children. The affectionate mother, while clasping her suffering infant in her arms, will anxiously listen for your footsteps in the hope of having it snatched by your skill from an early grave. Thus, day by day, you are to deal with the most confidential, the most important, and the most sacred interests of man. Let me entreat each one of you, then, in the prosecution of your professional career, not only to cultivate the highest degree of familiarity with every branch of medical science and art, but also a mental discipline, which will enable you to use the facts and materials with which you become familiar, with the highest degree of promptitude and skill, and a moral integrity that no temptations can swerve. If you do these things faithfully, when you go out from these halls, your lives and acts will constitute the most efficient support for your Alma Mater, and the world will be better and happier for your living in it.

